# A step-by-step guide to creating an academic surgery interest group: Review article

**DOI:** 10.1016/j.amsu.2021.102688

**Published:** 2021-08-05

**Authors:** Gonzalo Domínguez-Alvarado, Karla Villar-Rincón, María Castillo-Miranda, Angie Quintero-Díaz, Angie Ramírez-Rangel, Ivan David Lozada-Martínez, Luis López-Gómez, Maria Paz Bolaño-Romero, Sabrina Rahman

**Affiliations:** a Grupo de Innovación e Investigación Quirúrgica, Universidad Autónoma de Bucaramanga, Bucaramanga, Colombia; b Future Surgeons Chapter, Colombian Surgery Association, Bogotá, Colombia; c Semillero de Innovación e Investigación en Cirugía, Universidad Autónoma de Bucaramanga, Bucaramanga, Colombia; d Medical and Surgical Research Center, University of Cartagena, Cartagena, Colombia; e Colombian Association of Obesity and Bariatric Surgery, Bogotá, Colombia; f Department of Public Health, Independent University-Bangladesh, Dhaka, Bangladesh

**Keywords:** Interest group, Medical students, Medical education, Surgery, Academic surgery

## Abstract

An academic interest group in medicine is defined as a collective that can be made up of undergraduate students, residents, and/or teachers, who share as an object of interest the advancement and growth of a specific area of medicine. It is organized to carry out extracurricular academic and research activities. It is essential to stimulate participation in these interest groups, which allow the personal and professional growth of their members, being a tool that promotes and provides better opportunities for entry as candidates for graduate studies. The *American College of Surgeons* is one of the largest medical scientific societies with the participation of undergraduate students with an interest in surgery. This society mentions the benefits of participating in interest groups in surgery, specifically, it highlights the importance of contributing to these when looking for a surgical specialty, because they address issues such as: what is the mentioned specialty itself?, what are the details about the application process for each surgical specialty?, these groups can provide information on different residency programs, lifestyle benefits, and/or cons; in addition to improving practical skills through surgical technique workshops or various organized activities. Based on the above, the objective of this manuscript is to design a Step-by-step guide for the creation of a surgical interest group, in order to encourage participation by medical students, residents, and teachers in the research and academic field.

## Highlights

• An academic interest group in surgery reinforces skills and knowledge.
•An academic interest group in surgery can be coordinated by undergraduate students.
•Academic interest groups in surgery generate national and international knowledge networks.

## 1 Introduction

An interest group in medicine is defined as a collective that can be made up of undergraduate students, residents, and/or teachers, who share as an object of interest the advancement and growth of a specific area of medicine. It is organized to carry out extracurricular academic and research activities. It is essential to stimulate participation in these interest groups, which allow the personal and professional growth of their members, being a tool that promotes and provides better opportunities for entry as candidates for graduate studies [1]. This is the reason why, in recent years, they have become very important in medical faculties, with a notable increase in the number of groups in large institutions with potential scientific participation [[2], [3], [4]].

The American College of Surgeons is one of the largest medical scientific societies with the participation of undergraduate students with an interest in surgery [1]. This society mentions the benefits of participating in interest groups in surgery, specifically, it highlights the importance of contributing to these when looking for a surgical specialty, because they address issues such as: what is the mentioned specialty itself?, what are the details about the application process for each surgical specialty?, these groups can provide information on different residency programs, lifestyle benefits, and/or cons; in addition to improving practical skills through surgical technique workshops or various organized activities [1].

Both the organization of these groups and the activities carried out, significantly improve basic and clinical aspects in surgery, being a plus for those focused on surgical specialties [4,5]. Currently, despite the benefits of interest groups, there is little literature on this topic. Based on the above, the objective of this manuscript is to design a Step-by-step guide for the creation of a surgical interest group, in order to encourage participation by medical students, residents, and teachers in the research and academic field in surgery.

## 2 Methods

A non-systematic review of the literature was carried out in PubMed and Science Direct databases, using the keywords “Interest group”; “Surgery”; “Research” and “Medical Student”, as well as synonyms, which were combined with the operators “AND” and “OR”. The search date was carried out until March 2021. Original studies, reviews, letters, and comments were included. The only exclusion criterion was the unavailability of the full text. Finally, 28 articles were included. Additionally, a reverse search and a direct search in the google search tool was performed to find information on international scientific societies with formalized interest groups in surgery and other clinical sciences. A total of 12 references were obtained from this search, using a total of 40 references.

### 2.1 Benefits of interest groups in medical schools

Although basic principles of research methodology are taught in medical schools, there is a minority group of students who wish to deepen their knowledge and apply it in the conduct of research [5]. In contrast, there are few students who, at the time of their undergraduate studies, are aware of the benefits that research generates for the construction of their professional future and the future of the academic community because medical research has led to the development of new or improved surgical techniques, a better understanding of the cause-effect relationship of diseases, detailed descriptions of hereditary diseases and knowledge of their genetic components, identification, and treatment of new or emerging infectious diseases, among many more achievements [6.7].

If we take into account that an interest group is made up of students, residents, and/or teachers (medical specialists) who have a common interest, in which multiple activities are proposed in order to increase their own knowledge, research, and even, to identify and propose new strategies for a specific branch of medicine; we can then deduce that each member of the interest group will have the opportunity to substantially improve their skills in the practical and investigative area of the specialty they want to develop, through self-interest [5].

Likewise, these groups foster an academic and innovative environment in which the members will be able to improve their medical judgment against the different clinical scenarios based on good practices, enhance the development of surgical skills through activities that allow an approach to the specialty, by generating spaces where students and teachers exchange knowledge and expose their own experiences in order to correct shortcomings and/or improve the practical exercise of each member [6]. This improves confidence, allows for more up-to-date evidence-based approaches with fewer complications and much more effective success rates, strengthens the relationship between colleagues, and facilitates the creation of national-international collaboration networks, promoting future projection [8,9].

Therefore, it is necessary for these interest groups to have the support of different institutions, which must provide access to resources for the development of activities (classrooms, places of practice, knowledge, personnel, money, and technology) [7]. In this way groups can benefit from inter-institutional agreements, allowing them to create connections with national and international scientific societies, and thus work together, which allows the qualification of the resume of each participant and benefits to a great extent those doctors aspiring to a medical residence [6,9,10].

### 2.2 Impact of interest groups in academic surgery

Research is a high-impact topic in the field of medical education [9]. Unfortunately, this is a topic that is not usually encouraged at the beginning of the career, being unaware of the positive repercussions that this would imply in the future of any undergraduate student. Likewise, it is of great importance to participate in scientific publications that demonstrate that the student has an interest in innovating on problems in medicine and public health. A study conducted in Germany found that 28% of medical students were authors of an institution's publications, being first authors only in 7.8% of the articles, however, this was a motivating factor for students to continue their research careers [10].

In addition, it should be noted that researching during undergraduate studies and publishing the results, have a noticeable influence later on aspects such as job promotions, a comfortable salary, academic recognition, and scientific reputation. These aspects make a significant difference, important for acceptance in a postgraduate course [11]. Although some medical students are aware of the benefits, they can gain from devoting more time to research, a very small group dares to develop this path, as many think it is very difficult or something that is not directly related to doctors [12].

Sánchez-Duque et al. [13] conducted a descriptive study that consisted of doing a virtual course for medical students in Latin America, on high-impact scientific writing and publication, in which 897 students participated. They found that only 141 students passed the course and were taken as a sample study. From the sample studied, it was found that only 19.15% (n = 27) had made scientific publications, which from the beginning shows that the number of publications in which medical students are included is really low [13]. Among the results, it was evident that 64 students (45.39%) rated as good the level of training they receive in their university on research, but 102 (72.34%) students criticized the process of advice and motivation on the part of their teachers, thus, it could be concluded that the main reason why the study population did not investigate was the lack of advice [13]. On the other hand, 117 (82.97%) students said they liked research and therefore wanted to learn more from it.

This result justifies the creation of interest groups in which research is enhanced arising from personal interests. In this way, if there is an adequate articulation between a suitable accompaniment together with the student's inclination for research, better results will be obtained in the future [13]. Finally, the study demonstrated the importance of belonging to some kind of scientific society or research group, as 107 students (75.89%) referred to the key role played by their association with student societies and their association with research incubators to promote their research skills. Thus, belonging to a scientific society of medical students, it was significantly associated with having a greater scientific production in both the number of publications (p < 0.05) [13].

Waaijer et al. [14] studied whether undergraduate scientific publications are associated with post-graduation publication. For this, a follow-up was carried out to 4145 professionals 6 years after their graduation. It was evident that students who had published during their undergraduate studies were 1.9 times more likely to publish (RR 1.90; CI: 95% [1.76, 2.05], p < 0.001), published more articles, and had a slightly greater citation impact after graduation [14].

Al-Busaidi et al. [15] evaluated the effects of scientific productivity of medical students by examining the number of postgraduate publications in indexed journals in PubMed®, completion of higher academic studies, and achievement of the rank of faculty after graduation [15]. It was evident that compared to the control group, students who published were more likely to obtain a higher degree (55.1% (n = 27) compared to 31.6% (n = 31), and to obtain an academic position in a faculty (OR 2.90; CI: 95%: 1.01–8.30; p = 0.047) [15]. Finally, the study showed that participation in research activities during medical school is associated with subsequent academic success [15].

Ball & Harvey [16] expose the challenges that make the surgical research field increasingly less actively involved, given the omission of a constant emphasis on the importance of research productivity for academic training. Within the identified barriers, there was a reduction in both formal and structured support in some surgical departments/sections, difficulty in identifying and involving experienced research mentors, and a less directed effort towards research [16]. At the university level, on the other hand, the requirements for project approval are annoying and there is less and less access to funding from local and national bodies, which represents greater obstacles to success. The situation is very worrying, because the National Institutes of Health of the United States, has recorded that only 1.5% of doctors are dedicated to this branch, a figure that they rank as surprisingly low [17]. Based on the above, it is imperative to create strategies and spaces that arouse research interest in the early stages of the medical career [18].

All of the above points out the great opportunity that establishing interest groups in the various medical specialties in medical schools represents. In the case of surgery, formal research programs that include medical students can be optimized by improving the recognition of the student's effort, promoting student-mentor interaction, and allowing students the option to increase the duration of the research experience. The development of these skills during the undergraduate will facilitate the permanence of the professional in these activities during the future, helping him to reach a higher level of education and recognition.

### 2.3 How to create an academic surgery interest group?

The first Step in creating an interest group is to have the motivation and the willingness to comment, invite and involve other students and professionals who share the same interest, who agree to establish a role and order in the group, that allows the follow-up of the activities and those who also contributed their knowledge and/or experience in surgery. A model proposal for the creation of a surgical interest group is as follows:

Step One Designing the Group's Administrative Structure

The administrative model of an interest group depends on the number of members since it must be considered according to the objectives of the group so that it provides organization and allows good communication between the members. In the first place a temporary board of directors must be assigned, who will play the role of leaders, for this reason, it must be a mixed group (equity between sex and grades: students/residents/teachers). They will also represent the group and establish direct communication with the institutions. Following a hierarchical model, at least one student coordinator and one teacher coordinator should be chosen, depending on the size of the membership. These students will be in charge of the communication between the group, and the teacher coordinators will be the facilitators of all the processes. A treasurer will also be chosen to manage resources, mainly fund-raising [5].

**Fig. 1:**
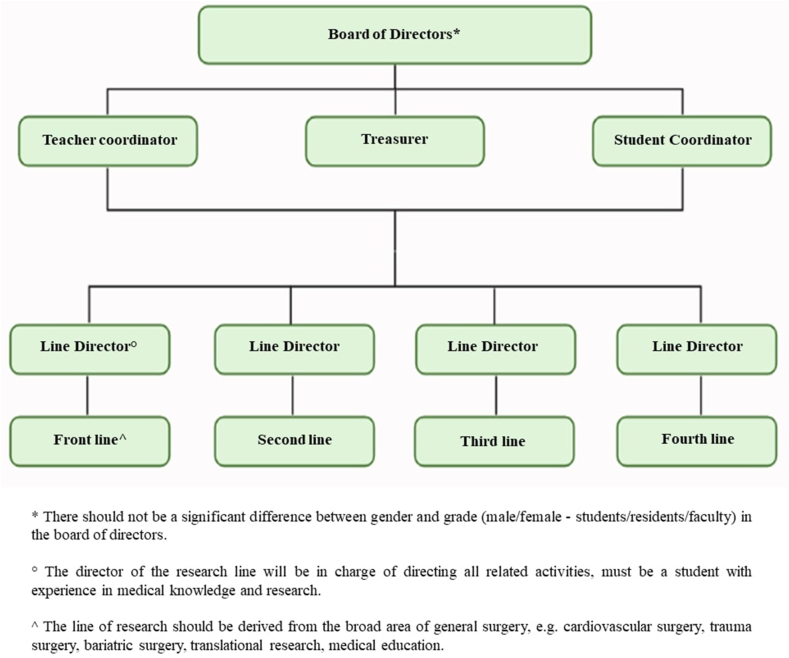
Administrative structure model of an interest group.

Finally, it is important to define the directors of the research lines in surgery that will be addressed in the interest group. Based on these lines, subgroups will be established to follow up the research work of each line, and to be able to evaluate the fulfillment of the goals of the group in general. The administrative structure may vary according to the objectives set; however, it can be based on a general structure (Fig. 1).

Step Two Acquiring Institutional, Academic, and Research Support

Before submitting the interest group proposal to the higher education institution, to obtain an official endorsement, it is important to specify the benefits that the interest group can contribute both to the institution and to the personal training of each member. Besides, it is important to consider that the permanent support of the institution will be necessary, to access classrooms and other resources from the institution, including among these, the different agreements that it has with hospitals or clinical practice sites.

On the other hand, it should be borne in mind that one of the most important objectives when participating in an interest group is to generate new inter-institutional and international connections, so the full support of the university will facilitate the creation of such connections.

Step Three To Raise the Research Branches to Develop

**Fig. 2:**
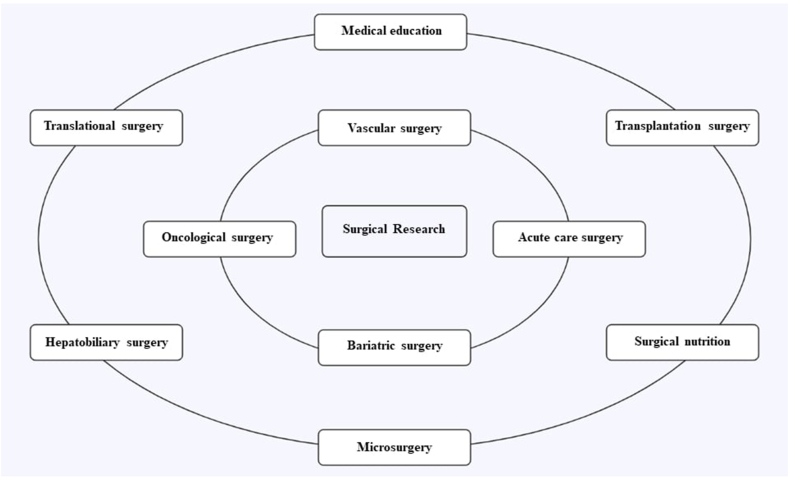
Summary of research areas to consider in a surgical interest group.

It is essential to establish which branches of research will be developed in the interest group and to specify how each will be covered. For this purpose, it is very useful to establish subgroups for participants to opt for the research line that most motivates them (Fig. 2).

Step Four To Establish a Schedule of Academic and Research Activities

To meet the objective and carry out the proposed goals within the interest group, a schedule of activities should be made. An example may be the following:

1. Once per week, each branch of research must hold a virtual or face-to-face academic meeting to follow up on its members and work.
2. Once per month, the entire interest group must hold a meeting to evaluate the results and monthly progress, to propose the following objectives
3. These meetings must respect the members' time and space. In addition, a message will be sent in advance setting the date, time, and general purpose of the meeting.
4. Reviews of topics in surgery or critical reading clubs should be carried out once per week, as these contribute to strengthening theoretical, methodological, and scientific writing knowledge. It should be led by a moderator with leadership and experience in the subject.
5. Theoretical and practical workshops on surgical skills and approaches should be established.
6. Local academic activities such as lectures, round tables, or talks should be organized in order to strengthen the critical skills of students and to strengthen interdisciplinary knowledge for clinical practice.
7. Clinical teaching-resident-student volunteer activities should be encouraged in public-private health institutions, as this opportunity will provide experience in working with patients and improving skills.

Step Five To Open Calls and Select Potential Members

To achieve the success of the interest group, it is necessary to carry out the mentoring exercise with those interested. That is why it is of vital importance to open calls once every year in search of potential members who contribute to the exponential growth of the group. A face-to-face meeting can be held to explain the dynamics, opportunities, and benefits of the group, as well as the duties acquired once they are accepted. For this, the use of digital marketing has played an important role today, social networks, such as WhatsApp, Twitter, Facebook, and Instagram [[19], [20], [21], [22], [23], [24], [25], [26]], can be used to welcome and attract the attention of new members [27,28].

Step Six To Give an Introduction to Mentoring and Research Methodology

Semi-annual training should be conducted, as well as group tutorials with epidemiologists and senior researchers with experience in research methodology, bibliography search, evidence-based medicine, and writing, providing tools and incentives to strengthen skills and knowledge, making known the benefits of these skills in professional life and the importance in clinical practice [5]. A very important aspect to take into account when publishing a scientific article or participating in a scientific event as a result of the work of the interest group is the authorship of the medical students. Considering that this is an academic exercise, it is necessary to be flexible with the authorship of students, respecting the Contributor Roles Taxonomy (CRediT) [29]. Therefore, if possible, they should be given the opportunity to work in the different roles established, so that they aspire to be first authors in any type of publication, which encourages them to continue contributing to the production of new knowledge and research.

Step Seven Define Group Roles and Specific Goals

To be able to have active control and promote group productivity, each member must be clear about their role and how they can contribute to the growth and improvement of the overall team [5].

Taking into account the pre-established hierarchical order:

1. The coordinating teachers will be responsible for introducing topics of current interest in surgery, based on their daily experience, in addition, they will carry out constant reviews of what has been produced.
2. Student coordinators will have the function of verifying the fulfillment of the tasks established for the different members of the research branches.
3. Finally, the directors of the branches will become the leaders of each subgroup, where they will carry out the active accompaniment to answer questions and evaluate the evolution of the members.

Step Eight To establish permanent support among higher-level students, residents, or teachers with leadership attitudes

It will be necessary to create an educational environment in which teacher and student coordinators maintain permanent contact, to ensure the continuous development of the research attitude [30,31]. Accompaniment will be crucial to make improvements, correct mistakes, support new ideas and clarify doubts. These elements will enhance the participatory quality of the interest group. Good teamwork can contribute to both strategic improvements and operational improvements, together with the commitment of the group, can be a potential source of innovation that will only be exploited if there is good leadership from the coordinating members [[32], [33], [34]].

Step Nine: Use social media management to disseminate academic content, achievements of the group and its members.

In recent years, the rise of social networks has become one of the most influential phenomena in the communication and dissemination of information. For this reason, it is essential to take advantage of all kinds of electronic tools to reach more professionals and students in general who share the same interests [35].

To accomplish this Step, we choose to:

1. Create social networks such as Instagram, Facebook, Twitter, and YouTube channel [[19], [20], [21], [22], [23], [24], [25], [26]].
2. Set goals with defined schedules to disseminate the information so that there is an active production of content.
3. The content may include scientific publications by members, lectures by teachers or students on topics of interest, advice on conducting research, the benefits of being part of the interest group, and even questionnaires, interactive trivia, or surveys.
4. Include contact numbers to facilitate access to interested persons who do not reside locally.

Step Ten Objective and Organized Reassessment of Group Goals

To evaluate the goals of the research group objectively and in an organized manner, these will be carried out periodically [4]:

1. To establish a time limit for meetings of 90 min or less for a better response from participants.
2. Registration with names, surnames, and email at each meeting to bring the attendance of the participants at each meeting.
3. General evaluation surveys of the group's academic and research activities.
4. Teacher evaluation surveys.
5. Evaluations of the topics viewed over a period of time.
6. Conferences with subspecialists of interest that allow a greater perspective of the specialty for the participants.
7. Detailed review of research work in each area.
8. Review of complaints or suggestions submitted by the different access routes of the research group.
9. To evaluate student and teacher compliance with the different programmed activities.
10. Meetings between teacher coordinators and student coordinators to analyze the functioning of the interest group, the progress made, and the organization of future activities.
11. Activities of simulation and discussion of clinical cases, also of the approach of research problems and club of journals to promote the learning of the students will be performed.
12. Permanent training programs will be established in research methodology, biostatistics, laboratory skills, bibliographic search, research ethics, critical reading, writing, and scientific publication.

Step 11 Strengthen national and international contacts in surgery

To strengthen knowledge and acquire greater tools to address patients; partnerships should be established with internationally recognized societies such as the *European Surgical Association* and the *American College of Surgeons*, to provide all participants access to specialized journals, international congresses, and conferences, as well as to strengthen teaching skills and inter-institutional relations. Other scientific societies with student participation include the *Latin American Federation of Surgery* (FELAC) [36], the *European Society of Thoracic Surgeons* (ESTS) [37] (which developed the Uniportal VATS interest group [38]), the *Society for Vascular Surgery* (SVS) and its interest group (V-SIG) [39] and the *Association for Surgical Education* (ASE) [40].

The ESTS Uniportal VATS Interest Group is a community of thoracic surgeons with a common interest in the technique of video-assisted thoracic surgery, which has had a major impact on major lung resections and the way surgeons treat intrathoracic disease. Such an interest group serves as a learning nest for all those students and physicians interested in thoracic surgery [37,38]. Since its inception, FELAC's objective has been the promotion and improvement of research, teaching and practice of surgery, as well as the promotion of fraternal coexistence of Latin American surgeons. This society periodically organizes events with the participation of members of all surgical associations in the region, allowing the attendance of students and physicians with particular interest in surgical sciences [36].

Likewise, the SVS is a society composed primarily of vascular surgeons, which seeks to promote excellence and innovation in vascular medicine through education, prevention and promotion, research and public awareness. Its interest group (V-SIG) aims to provide students with early exposure to surgery, help students become involved in research activities related to vascular medicine topics early in their medical careers, and gain valuable career guidance [39]. Hand in hand, the ASE notes that for the medical student, a surgical interest group generates potential advantages such as the opportunity to explore professional issues, gain greater exposure to the breadth and scope of surgical fields, identify research opportunities, develop mentoring relationships, and obtain information about internship experiences and residency programs. In addition, surgical interest groups are a strategy for conveying the positive and exciting aspects of a surgical career, and ultimately may result in increased recruitment of students into surgical residencies [40].

Likewise, agreements should be sought with different clinics and hospitals that allow access for teachers and students to the different simulation laboratories, so that this is a propitious scenario in learning. Besides, this will improve relations through participation in protocolary events of different institutions, establish agreements with institutions or pharmaceutical industries that contribute economically to the development of activities [10,18].

## 3 Conclusion

Surgical interest groups are an indispensable strategy for the development of academic surgery. They allow professional and research growth in medical schools, increasing the skills of both students and teachers. They create and strengthen inter-agency expertise networks, and facilitate targeted learning for postgraduate access. This academic tool should be put into practice in all medical schools, not only focusing on surgery, but on all medical specialties and different fields in health sciences.

## Provenance and peer review

Not commissioned, externally peer reviewed.

## Please state whether Ethical approval was given, by whom and the relevant Judgement's reference number

It is not necessary.

## Please state any sources of funding for your research

None.

## Author contribution

All authors equally contributed to the analysis and writing of the manuscript.

## Please state any conflicts of interest

None.

## Research registration Unique Identifying Number (UIN)

Name of the registry: Not applicable.
Unique Identifying number or registration ID: Not applicable.
Hyperlink to your specific registration (must be publicly accessible and will be checked): Not applicable.

## Guarantor

The Guarantor is the one or more people who accept full responsibility for the work and/or the conduct of the study, had access to the data, and controlled the decision to publish. Please note that providing a guarantor is compulsory.

Sabrina Rahman. Department of Public Health, Independent University-Bangladesh, Dhaka, Bangladesh. sabrinaemz25@gmail.com.
